# Professional Intercourse

**Published:** 1859-10

**Authors:** James Taylor


### DENTAL REGISTER OF THE WEST.

Vol. XIII.] OCTOBER, 1859. [No. 4.

### Original Essays and Communications.

### PROFESSIONAL INTERCOURSE.

A PAPER READ BEFORE THE AMERICAN DENTAL CONVENTION.

BY JAMES TAYLOR, M. D. D. D. S.

Mr. President:—In the consideration of this subject we might ask, What is there in professional intercourse which should distinguish it, from that intercourse which should mark the conduct of one gentleman towards another? Every gentleman has a right to expect, and may demand that respect and deference which the proprieties of life and the social customs of society impose.

The very term *gentleman* means a great deal: it is composed of two words, GENTLE, mild, not refractory, courteous, kind, considerate, obliging, not overbearing, or stormy, tyrannical, arbitrary, dogmatical; MAN, the masculine of the human race, a being endowed with reason, judgment, wisdom, skill. Every man should be, a *noble man* the "noblest work of God," in all the amenities of life, he constantly gives what he feels in his nature he should receive. In all his acts, integrity, a strict regard to truth, and the right of others, mark him as the gentleman. But the very question pre-supposes something additional to all this, and yet not much can be added; for we take it for granted that no member of a profession, like our own, can be known and recognized as a brother, without a clear and just title to that of a *gentleman.* In all professions, however, there are direct professional obligations, which do not exist in the ordinary intercourse of life; 'tis true these are few, and might, perhaps, with strict propriety be classed with the others. Allow me to draw attention to one or two.

There is a duty one owes his profession, and in the exercise of this duty he has a double intercourse imposed upon him,—that with the different members of the profession, and that with the public. Every man in assuming a liberal profession, imposes upon himself a two-fold obligation—first, a duty to that particular department of science, and second, a duty to the public who, of a right, look to that science for benefit. We are *mere recipients for the transmission of knowledge.* All the little details of all the great facts or principles which make up our quantum of knowledge are not our own, but belong to the profession we have chosen.

It is this great principle which distinguishes all the liberal professions; throw aside this principle, and we can not, we dare not claim that distinction. Every intelligent and liberal professional man would laugh at us for such an assumption. We must come on to this broad platform, great, grand and benificent, "Freely we have received, freely give."

But let us analyze this question a little closer. We have chosen a profession, the object and aim of which is to arrest and repair the ravages of disease: a *noble profession* it is. Take all the arcana of human knowledge, and sift therefrom that which will most conduce to the happiness of our race, and medical science stands pre-eminently at the head. What is dental science but a speciality of medicine, which grasps in its embrace every principle on which that is based? Does medical science come to us rich in the lore of antiquity, and still made more rich by the great and good of our own age?

Does it come to us ladened with the trophies of the most self-sacrificing, untiring, and indefatigable benefactor of our race? Does it come with "healing on its wings," a balm for every wound in its hands, and tears of mercy in its eyes, and yet *we* refuse to contribute our mite to its onward march in the cause of humanity? Does not rather the terms of our enlistment demand, at our hands, every accession that we make, every additional fact in science we may glean to enrich her treasures, every ray of light we may eliminate by our research to impel her onward progress?

In this great conquest over disease, we are not left to consult our own self-aggrandisement. Each man *should feel* that above and overreaching all this, there is a nobler and a higher aim, an aim, which, while it brings competence and reward, stretches beyond, even to elevate and advance our profession itself. Every member of the profession should feel that to elevate dental science is to elevate himself.

That his honor, his reputation, his very manhood, what he *is*, and what he *is* to be, are wrapped up in his calling. What he owes to himself, this, and far more, he owes to his profession. He can not say with Cain, Am I my brother's keeper? for he has no right to degrade his brother's calling. He *may* claim the right to disfranchise himself, but he has no right to disfranchise his brother or his profession.

Let it then be remembered, that he who seeks isolation from responsibility, who would throw off all the weighty obligations, which an intelligent and liberal profession impose, must step aside from its duties; nay, he must seek none of its honors, none of its rewards, not even its mantle as a winding-sheet.

This one fact is then self-evident, that it is the first great duty of every member of the profession to seek its highest elevation, to stand up for its reputation. We would take no narrow view of professional intercourse, but we would have our intercourse high-minded, liberal and intelligent: it means far more than the every day interviews with our brethren.

We would commune and hold intercourse with our science every day of our life. We would seek her requirements, consult her good. We would lay as tributaries at her feet every department of knowledge which could aid in her progress.

It is the duty, then, of every member of the profession to see that the standard of dental attainments be placed as high as possible; that, not only should his own progress in knowledge keep pace with the demands of the science, but that every new candidate for her honors should not pass the portal of her sanctuary, until prepared to meet the responsibilities he assumes. There is no need of mincing the matter; the profession as it is, has *now* in its own hands the future repute and character of dental science. We can not in honor recede, we must in honor go on. A survey of study may frighten many from the field, who but for this would enter; yet the brave will enter, and achieve most honor, where the timid hesitate and draw back. It should be known, the sooner the better, that it is no child's play to pass through the various studies required for the dental practitioner. Take all you dare, from the regular study of medicine, and then add the absolutely requisite for the dental student, and the difference is small. Dentistry demands much that medicine may do without. It is the duty, then, of every member of the profession to do every thing in his power to elevate and advance the standard of dental attainment. He comes down to the ordinary intercourse of life, and as he may meet his brethren, it is not a turn of the key on this thought, a locking up in his vault, an important principle he may wish to unravel at some future day himself—but his pride of the profession, seeks the first aid he may get, that the principle may be tested. How many important improvements are held back, for the want of united effort? How many wonderful discoveries are working sore and sad disappointments to the inventors, because covered up from the tests which science might and would apply, if revealed?

Every professional man *should* feel, that every new thing, whether in principle or practice, can not be firmly established, until it has passed the ordeal of the profession itself.

Time with her "relentless *tooth*," and experience with her sad realities, make terrible havoc sometimes among our most cherished air castles.

Let but our professional zeal outstrip our selfish prudence, and the rapid advance in our science would startle and amaze the most incredulous.

The vast amount of useless research, if guided aright by the known principles of science would accomplish much in the way of improvement. The time, talent and skill which have been expended on the idea of a perpetual motion, might have renovated every mechanical and artistic trade in the world; thousands of times it has been accomplished but for one *little* thing, yet that *little* thing is as the mountain itself, it can not be moved.

In our professional intercourse, there is one duty we owe to the profession and the public at large, which demands more than a mere passing notice; this is the sentiments we may inculcate in relation to dental education. Time was, when many honestly thought, that a few weeks, or months at most, was time sufficient to master all the details of dental practice; sad experience has taught us a lesson on this subject; times have changed, public sentiment has changed, and if dental science has not changed, it has progressed. Things have changed about our office, things have changed in our laboratory, and if we change not, over our doors will be written, "OLD FOGY." It becomes every member of the profession to see to it, that no student leaves his office without thorough preparation, not only in the mechanical detail of artistic dentistry, but the general principles of medical science on which the profession can alone stand. Long observation has convinced us that theoretical principles neglected before practice begins, but seldom receive much attention thereafter, while mechanical skill, being an every day necessity, must be attended to.

The demands of life, or the glitter of the fee itself, closes on the perspective, and hides all but the daily duties of the man. There is a period of life which emphatically belongs to pupilage, and we doubt not, that observation will convince any one, that if in the hurry to assume the responsibilities of practice, this is neglected or cut short, the individual is injured professionally as well as pecuniarily. There are laws which work out certain results, almost as surely in morals and in the practical details of life, as in astronomy itself. These laws appear to be almost immutable, none of us imbibe a great amount of professional knowledge by intuition. It comes to us through two channels—the brain and the hands—the latter are an indispensable requisite to the dental practitioner, they need a great deal of careful education, but still they can't say to the brain, " I have no need of thee."

The brain must be the moving power; neglect this great propelling principle, the motive force which directs and controls every manipulation of our art, and you might as well place instruments in the hands of wood-choppers.

We have individuals enough now—" whether in the body or out of the body, we cannot tell"—who have been moving around for years, catching an item here, and picking up one there, to fill all the dental colleges in our land. There is no want of *hands*, we can not say of *brains*, but they can't get both to work well together, they will not agree; the hands can not do what the brain wills, and the poor brain has never been taught to will aright.

Mr. PRESIDENT, Are not these stubborn facts? We appeal to the good sense of every member of this Convention if these things are not so. Shall we be told, then, that there is no remedy? there is a remedy, and *you* my brother, and *you*, and *you*,—yes *we* have this remedy in our hands; let us have the moral courage to use it.

We do not propose the adoption of any resolution by this Convention on this subject, we know that resolutions thus passed would be of no avail, unless backed by the firm resolve of every member to carry them out. We do, however, hope to enlist your feelings so in this matter, that your acts will be far more effective than any resolutions.

We presume that if the question were now asked, what is the lowest term at which pupilage should be placed, the unanimous response would be, not less than two or three years; if so, carry it out, and let the profession through your intercourse with it, know your views on this subject, and the influence will be felt. We do not come before you with this subject in behalf of our Dental Colleges, yet if time, labor and money expended, would entitle any one to press such a subject, we might: but we do come with this subject, appealing to the good sense and judgment of every member of the profession, that they may meet its requirements in the light of true science.

Make of our profession a *trade*, and we leave it to-morow; not but that trades are respectable, and equally honorable, but that science would receive a wound we have not the courage to inflict.

Mr. PRESIDENT. In conclusion, we would not advocate that kind of professional intercourse which would sustain the disreputable conduct of a brother; in doing this we might and would degrade the profession itself. We would go as far as any man, as far as honor bright would allow, to sustain the right; yet we should all feel, that to go farther would deserve the execration of every true friend of the profession.

To be honest, we must be just. True professional courtesy requires that wherever we can, we should commend the operations of another; there is a vast amount of indiscretion on this subject, operations are examined years after they have been performed; the carelessness of the patient, or the progress of disease has changed the condition of the mouth very much, and because the operation has not proved as durable as desired, the whole is condemned. We can not tell all the circumstances of the case; there may have been difficulties in the way, the patient with the then sensitive condition of the teeth, may have been unable to endure all the pain necessary for the most thorough work.

What may prove a defective operation, may not always be for want of skill in the operator. Duty to our patient requires that we should do the very best we can, under the circumstances, for his good. If the case is such that no good can be accomplished, refuse at once to operate.

From our peculiar locality—being about in the centre of the world, and half way between the east and the west, the north and the south, we see almost daily, operations from under the hands of the most skilled in the world, yet time and circumstances have so changed the condition of things, that charity's mantle must be thrown over the whole. The fact is, there is not after all much perfection in this world, we like to see it, and are willing to praise it at any time, it is very mortifying, however, to find we have *crowed* too soon.

Mr. PRESIDENT, These younger practitioners do not know what professional intercourse *used to be.* But fear not, ye time-honored fathers, we draw over the past the curtain of oblivion, we have cast off the shackles, forged and riveted on in the dark ages, we now stand up in our professional freedom, exultant in victory, and shall ever rally to our cause the intelligent, the liberal, the noble and the brave, who battle for the advance of our science.

### DENTAL QUACKERY.

A PAPER READ BEFORE THE AMERICAN DENTAL CONVENTION.

